# Platinum-containing compound platinum pyrithione suppresses ovarian tumor proliferation through proteasome inhibition

**DOI:** 10.1186/s13046-017-0547-8

**Published:** 2017-06-15

**Authors:** Hongbiao Huang, Ni Liu, Yuning Liao, Ningning Liu, Jianyu Cai, Xiaohong Xia, Zhiqiang Guo, Yanling Li, Qirong Wen, Qi Yin, Yan Liu, Qingxia Wu, Dhivya Rajakumar, Xiujie Sheng, Jinbao Liu

**Affiliations:** 1 Key Laboratory of Protein Modification and Degradation, Department of Obsterics and Gynecology, The Third Affiliated Hospital, Key Laboratory for Major Obstetric Diseases of Guangdong Province, Guangzhou, Guangdong 510510 China; 2 Key Laboratory of Protein Modification and Degradation, School of Basic Medical Sciences and Affiliated Cancer Hospital of Guangzhou Medical University, Guangzhou, Guangdong 511436 China; 3 Guangzhou Institute of Cardiovascular Disease, the Second Affiliated Hospital, Guangzhou Medical University, Guangzhou, Guangdong 510260 China

**Keywords:** PtPT, Proteasome, Deubiquitinase, Epithelial ovarian cancer, Proliferation

## Abstract

**Background:**

Ovarian carcinoma is one of the most aggressive gynecological malignant neoplasms and makes up 25–30% of all cancer cases of the female genital tract. Currently, resistance to traditional chemotherapy is a great challenge for patients with Epithelial ovarian cancer (EOC). Therefore, identifying novel agents for EOC treatment is essential and urgent.

**Method:**

MTS assay was used to analyze the cell viability and proliferation of cancer cells. Flow cytometry was employed to analyze cell cycle distribution and cell apoptosis. Protein signaling pathways were detected by western blot and immunohistochemical staining. Nude mouse experiment was performed to test the in vivo effect of platinum pyrithione (PtPT).

**Results:**

PtPT is a chemically well-characterized synthetic complex of platinum that potently inhibits proteasome-associated deubiquitinases USP14 and UCHL5 activity and shows selective cytotoxicity to multiple cancer cells without damaging DNA. We found that PtPT significantly accumulated ubquitinated-proteins and suppressed the proliferation of multiple EOC cells. Additionally, PtPT induced G2 phase arrest and apoptosis in both A2780 and SKOV3 cells. More importantly, animal experiments showed that PtPT dramatically suppressed the growth of EOC xenografts without obvious side effects.

**Conclusion:**

These results suggest that through proteasome inhibition, PtPT significantly suppressed the proliferation of EOC in vitro and in vivo and could be developed as a novel agent for EOC treatment in the future.

## Background

Ovarian cancer is one of the most lethal gynecological malignancies in women, with more than 60,000 new cases reported annually in the United States and the European Union, and approximately 239,000 new cases worldwide were reported in 2012 [1, 2]. Due to the difficultly of early diagnosis and the hidden progression of the disease, most patients are diagnosed at the advanced stage of cancer, which carries an extremely poor prognosis [3]. Although the new era of targeted drugs has made some progress in ovarian cancer treatment, the antitumor efficacies of current therapies remain limited [4]. Therefore, it is urgent to develop novel and alternative strategies for epithelial ovarian cancer (EOC) treatment.

Proteasomes have already become effective therapeutic targets in some disease models such as inflammation, fibrosis, ischemia-reperfusion injury, and tumors [5, 6]. Cancer cells have been shown to depend on the ubiquitin proteasome system (UPS) more than normal cells do [7]. Therefore, proteasome inhibition has become an effective strategy for cancer therapy. Bortezomib is a classic 20S proteasome inhibitor (PI) approved by FDA for the treatment of multiple myeloma and mantle cell lymphoma. Inhibition of the proteasome with bortezomib was the first clinical validation of targeting the UPS in cancer therapeutics and has gotten a huge success. However, PIs have weak effect on solid tumors in humans [8]. Alternative proteasome inhibitors with different targets for proteasome activity sites were needed to make more satisfying effects on epithelial ovarian cancer (EOC).

Inhibition of proteasome-associated deubiquitinases (DUBs) has emerged as a promising anti-cancer strategy [9–11]. DUBs function to remove the ubquitin conjugates on protein substrates prior to their degradation and thereby regulating multiple cellular processes, which are highly associated with cancer development [12–15]. The human genome encodes approximately 100 putative DUBs, which are subdivided into six families according to their catalytic and structural features. USP14 and UCHL5 belong to DUBs and could be recruited and activated by 19S proteasome. Overexpression of USP14 or UCHL5 was observed in several carcinomas. Selective inhibition of USP14 and UCHL5 by b-AP15 or auranofin has been developed as a promising strategy against some carcinomas [16–18].

Cisplatin is one of the most effective drugs currently used for treatment of ovarian cancer, biliary cancer, lung cancer, etc. [19, 20]. However, continuous administration of Cisplatin to patients leads to severe side effects and the acquired or intrinsic resistance to Cisplatin, which gradually limits its use [21, 22]. We previously dedicated to identify a novel inhibitor of 26S proteasome-associated deubiquitinases USP14 and UCHL5. Our previous study revealed that PtPT, a chemically well-characterized synthetic complex of platinum, targets 26S proteasome-associated DUBs rather than DNA in the cell and thereby exerts safer and potent anti-tumor effects [23]. The current study aims to investigate the anti-tumor effects of PtPT on epithelial ovarian cancer and its underlying mechanisms.

## Methods

### Materials

Platinum pyrithione was dissolved in dimethyl sulfoxide (DMSO) to a concentration of 10 mM and stored at −20 °C, which was synthesized in our lab. Pan caspase inhibitor z-VAD-FMK was purchased from Enzo Life Sciences International, Inc. (Plymouth Meeting, PA). MTS assay (CellTiter 96 Aqueous One Solution reagent) was obtained from Promega Corporation (Madison, WI, USA). Penicillin, Streptomycin, and Cisplatin were from Sigma-Aldrich (St. Louis, MO, USA); Annexin V-FITC/PI apoptosis Detection Kit and cell apoptosis Rhodamine 123 Detection Kit were from Keygen Company (Nanjing, China). Antibodies raised against ubiquitin (Catalog #3936; 1:1000), Cdc2 (Catalog #9116; 1:1000), Cyclin B1 (Catalog #12231;1:1000), caspase-3 (Catalog #9662;1:1000), caspase-9 (Catalog #9508; 1:1000), caspase-8 (Catalog #9746;1:1000), PARP (Catalog #9532;1:1000), K48- linkage specific polyubiquitin (Catalog #12805;1:1000), K63- linkage specific polyubiquitin (Catalog #12930;1:1000), Bcl-2 (Catalog #15071;1:1000), Bax (Catalog #14796;1:1000), eIF2α (Catalog #5324;1:1000), Phospho-eIF2α (Catalog #3398; 1:1000), ATF-4 (Catalog #11815;1:1000), apoptosis-inducing factor (AIF) (Catalog #5318; 1:1000), and Cox-4 (Catalog #4844; 1:1000) were from Cell Signaling Technology (Beverly, MA, USA). Cleaved caspase-8 (Catalog #L0167; 1:1000) was from Assay biotechnology Company (California, USA); Cleaved caspase-9 (Catalog #BS7070; 1:1000), cleaved caspase-3 (Catalog #BS7004; 1:1000), and GAPDH (Catalog #BS60630; 1:1000) were from Bioworld Technology (St. Louis Park, MN, USA). Enhanced chemiluminescence (ECL) reagents were from Santa Cruz Biotechnology Inc. (Santa Cruz, CA, USA).

### Cell culture

Human ovarian cancer cell lines HO8910,OVCAR3, A2780, and SKOV3 were purchased from the American Type Culture Collection (ATCC; Manassas, VA, USA) and grown in Dulbecco’s modified Eagle’s medium (DMEM) supplemented with 10% FBS (Gibco, Carlsbad, CA, USA), 100 units/ml of penicillin and 100 g/ml of streptomycin, cultured at 37 °C with 5% CO2.

### Cell viability assay

Cell viability was assessed with the MTS assay as previously reported [24]. HO8910, OVCAR3, A2780, and SKOV3 cells were seeded into 96-well plates at a concentration of approximately 5000 cells/well in 100 μl overnight. After stimulation with various concentrations of PtPT or cisplatin (0, 2, 4, 6, 8, 10, 12 and 14 μM) for 24 and 48 h, 20 μl MTS was supplemented for another 3 h of incubation. Absorbance was read at 490 nm and IC_50_ values were derived.

### Flow cytometry analysis of cell cycle

Cell cycle analysis was performed as reported previously [25]. A2780 or SKOV3 cells were seeded in 6-cm dishes overnight in DMEM supplemented with 10% FBS, then treated with PtPT for 24 h, and the cells were digested by trypsin and washed twice with ice-cold PBS. The cell pellet was suspended with 70% ethanol at −20 °C overnight, then washed by PBS and incubated with 200 μg/mL RNase A at 37 °C for 15 min. For flow cytometry, 50 μg/mL propidium iodide (final concentration) was added for 15 min staining in the dark at 37 °C. Data was analyzed based on the distribution of cell populations at different phases of the cell cycle.

### Cell death assay

Apoptosis was assessed by flow cytometry using Annexin V-FITC and PI double staining as we previously reported [26]. After treatment with PtPT at various concentrations for 48 h, the cells were incubated in the dark for 15 min at room temperature with Annexin V-FITC followed by PI addition just before analysis. In addition, cells were submitted to Annexin V/PI staining in situ for observation by fluorescence microscopy.

### Mitochondrial membrane integrity measurement

The mitochondrial membrane potential of PtPT-treated and untreated cells were assayed by using rhodamine-123 staining as previously reported [27]. In brief, cells were treated with various concentrations of PtPT for 24 h and stained with 1 μM of rhodamine-123 for 30 min at 37 °C. Following the staining, the cells were washed with 4 °C PBS and then harvested for flow cytometry analysis.

### Western blot analysis

Western blot was performed as we described previously [28]. In brief, an equal amount of the total protein extracted from cultured cells was fractionated by 12% SDS-PAGE and transferred to polyvinylidene difluoride (PVDF) membranes. The blots were blocked with 5% milk for 1 h. Primary antibodies (1:1000) and horseradish peroxidase-conjugated appropriate secondary antibodies (1:5000) were used to detect the designated proteins. Blots were reacted to the ECL detection reagents and exposed to X-ray films (Kodak, Japan).

### Nude mouse xenograft model

Female Balb/c nude mice aged 5 weeks were purchased from Guangdong Animal Center and all animal protocols used were approved by the Institutional Animal Care and Use Committee of Guangzhou Medical University. The mice were housed in barrier facilities with a 12 h light dark cycle, with food and water available ad libitum. Approximately 1 × 10^6^ of A2780 or SKOV3 cells were inoculated subcutaneously in the left armpit of each mouse. After 5 days of inoculation, mice were treated with either vehicle (10% DMSO, 30% cremophor and 60% NaCl) or PtPT (7.5 mg/kg/day) for 23 days [23]. Tumors were measured every other day with the use of calipers. Tumor volumes were calculated as previously reported [29]. Tumor xenografts were removed afterwards, weighed, fixed, and stored. After 23 days of treatment, tumor xenografts were removed, weighed, stored, and fixed. All experiments were performed in accordance with relevant guidelines and regulations.

### Immunohistochemical staining

Formalin-fixed xenografts were embedded in paraffin and sectioned according to standard techniques as previously reported [29]. Tumor xenograft sections (4 μm) were immunostained using the MaxVision kit (Maixin Biol, Fuzhou, Fujian, China) according to the manufacturer’s instructions. The primary antibodies were against proteins as indicated. 50 μl MaxVisionTM reagent was applied to each slide. Color was developed with 0.05% diaminobenzidine and 0.03% H_2_O_2_ in 50 mM Tris–HCl (pH 7.6), and the slides were counterstained with hematoxylin. A negative control for every antibody was also included for each xenograft specimen by substituting the primary antibody with preimmune rabbit serum.

### Statistical analysis

All the experiments were performed at least thrice and were expressed as Mean ± SD where applicable. SPSS (SPSS, USA) was employed for the statistics. Unpaired Student’s *t*-test or one way ANOVA is used where appropriate for determining statistic probabilities. *P* <0.05 was considered statistically significant.

## Results

### PtPT suppressed cell proliferation of ovarian cancer cells

**Fig. 1:**
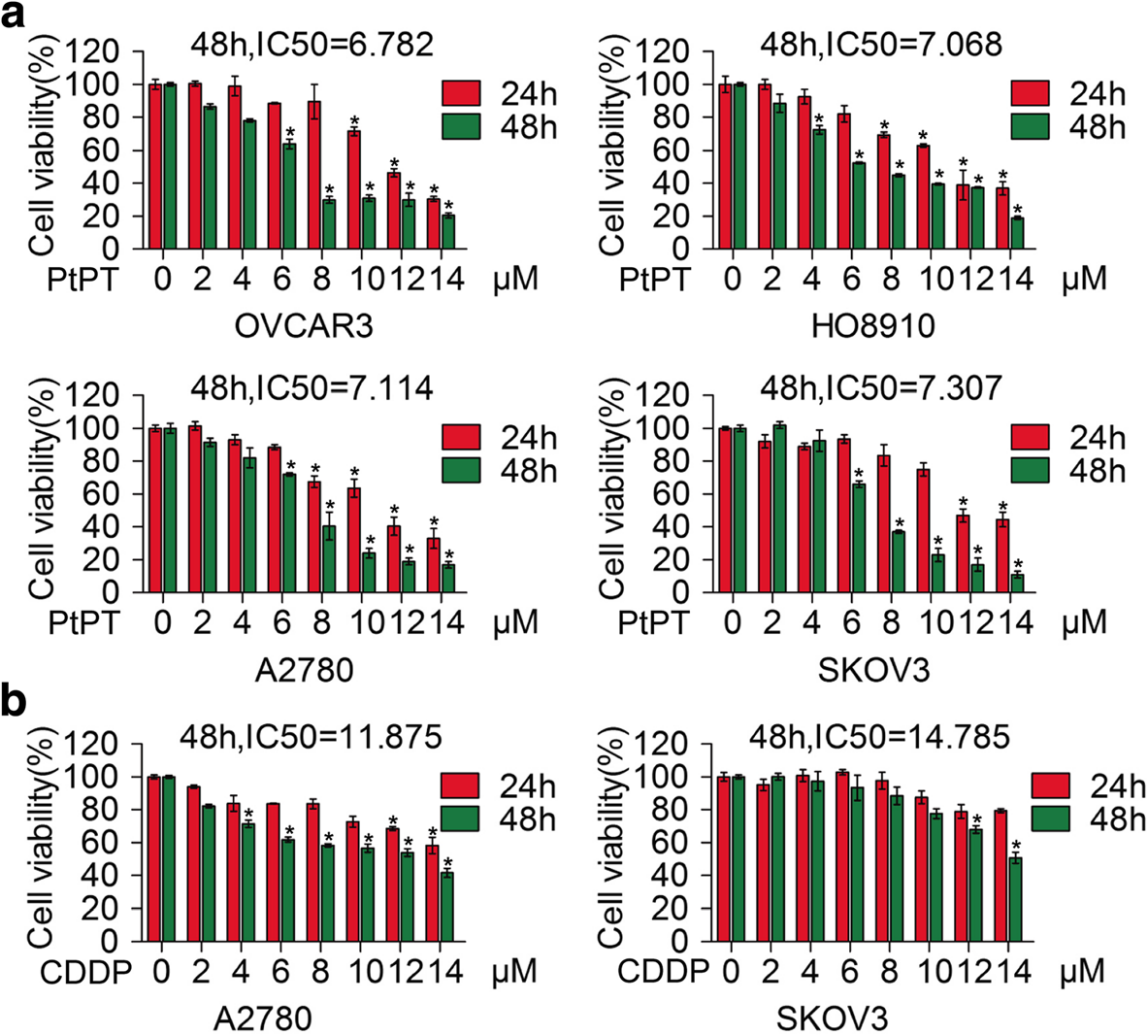
PtPT decreased viability in cultured ovarian cancer cells. **a** OVCAR 3, HO8910, A2780, and SKOV3 cells were exposed to indicating concentrations of PtPT for 24 or 48 h. Cell viability was detected by MTS assay. Graphs represent data from three independent experiments. Mean ± SD (*n* = 3); *P < 0.05. **b** A2780 and SKOV3 cells were exposed to indicating doses of CDDP for 24 or 48 h. Cell viability was detected by MTS assay. Mean ± SD (*n* = 3); *P < 0.05

In the attempt to evaluate the effects of PtPT on the growth of four human ovarian cancer cells, OVCAR3, HO8910, A2780, and SKOV3 cells were cultured and exposed to various concentrations of PtPT for 24 or 48 h and cell viability was detected by MTS assay. As shown in Fig. 1a, PtPT significantly reduced the cell viability in a dose- and time-dependent manner with the 48 h IC50 values of 6.782, 7.068, 7.114, and 7.307 μM respectively in OVCAR3, HO8910, A2780 and SKOV3 cells. To determine the antitumor efficacies of CDDP on EOC, we then investigated the proliferation of A2780 and SKOV3 cell lines exposed to indicated concentrations of CDDP using MTS assay (Fig. 1b). The results showed that exposure to increasing doses of CDDP over a period of 24 h and 48 h decreased the viability of cancer cells in a dose and time-dependent fashion but showed poor efficacy when compared with PtPT. Together, this finding suggested that PtPT possesses prominent antitumor activity in EOC.

### PtPT induces G2/M phase cell cycle arrest

**Fig. 2:**
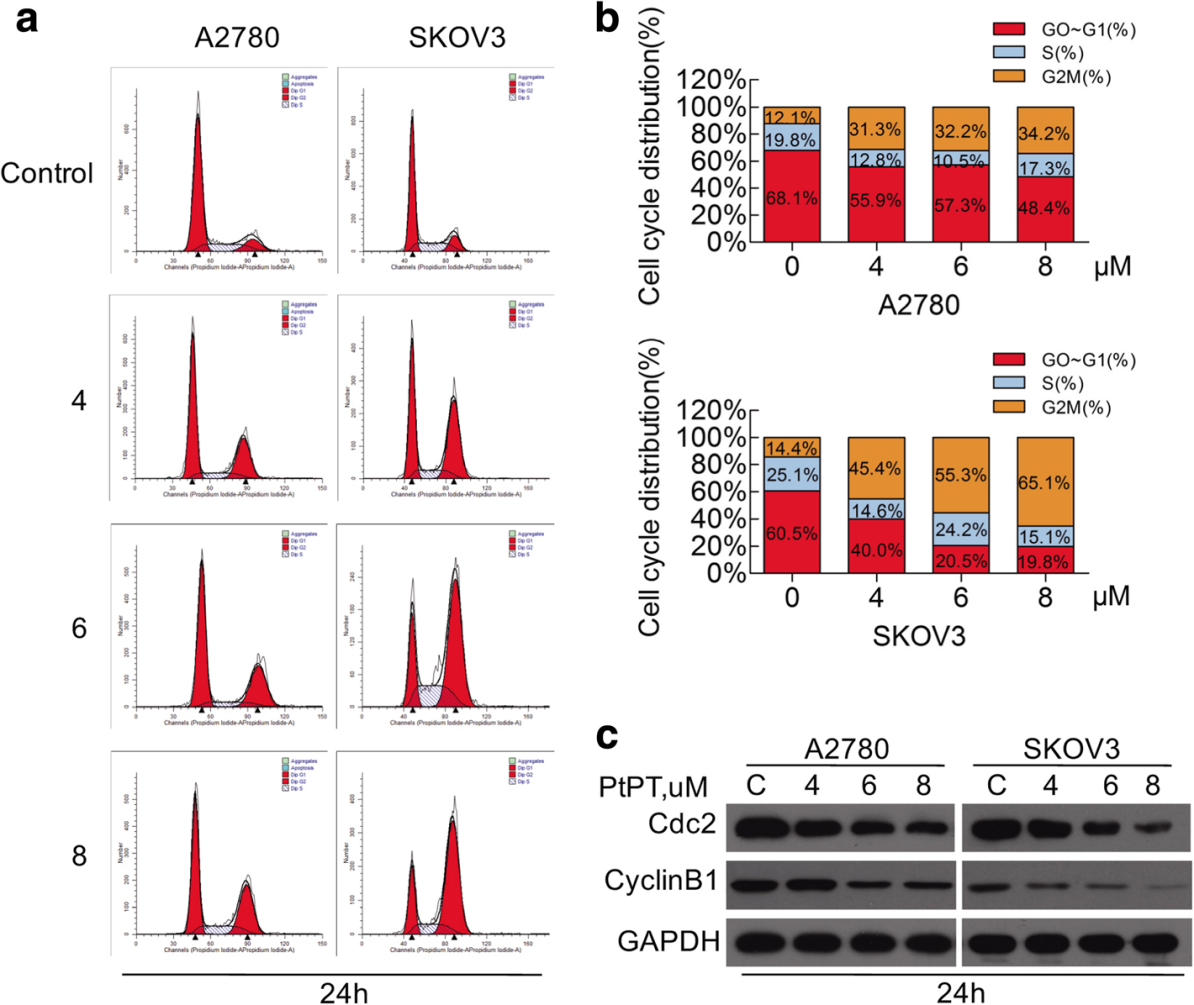
PtPT treatment induced G2/M cell cycle arrest in ovarian cancer cells. **a** A2780 and SKOV3 cells were treated with 4–8 μM PtPT for 24 h and subsequent to PI staining and flow cytometry analysis. Representative images were shown. **b** Graphs of the analysis were shown. **c** Proteins extracts from A2780 and SKOV3 cells exposed to indicating concentrations of PtPT were subjected to Western blot analysis by using antibodies against Cdc2 and Cyclin B1. GAPDH was used as a loading control

Cell cycle dysregulation contributes to the development and progression of EOC. Given that PtPT significantly suppressed the growth of EOC cells, we wonder whether the cell proliferation suppression by PtPT is caused by specific perturbation of cell cycle-related events. A2780 and SKOV3 cell lines were selected for further study. Cell cycle phase distribution of cells treated with PtPT (0, 4, 6, 8 μM) for 24 h were detected by flow cytometry. We found that A2780 and SKOV3 cells treated with PtPT resulted in a significant increase in the proportion of cells at the G2/M phase and a reduction in the proportion of cells at the G1 phases in a dose-dependent manner (*p* < 0.05) (Fig. 2a and b). The G2 phase percentage of A2780 ovarian cancer cells increased by approximately 22.1%, and the SKOV3 cells increased by 50.7% after 8 μM PtPT treatment for 24 h. These results together indicated that PtPT has the potential to induce G2/M cell cycle arrest on A2780 and SKOV3 cells. To investigate the underlying molecular mechanism by which PtPT arrested G2/M phase, we performed western blot analysis of G2/M-related protein expression levels in A2780 and SKOV3 cells exposed to the indicating doses of PtPT at 24 h. Figure 2c shows that PtPT dramatically downregulated Cdc2 and Cyclin B1 protein expression in a dose-dependent manner in both cell lines.

### PtPT induces apoptosis in A2780 and SKOV3 cells

**Fig. 3:**
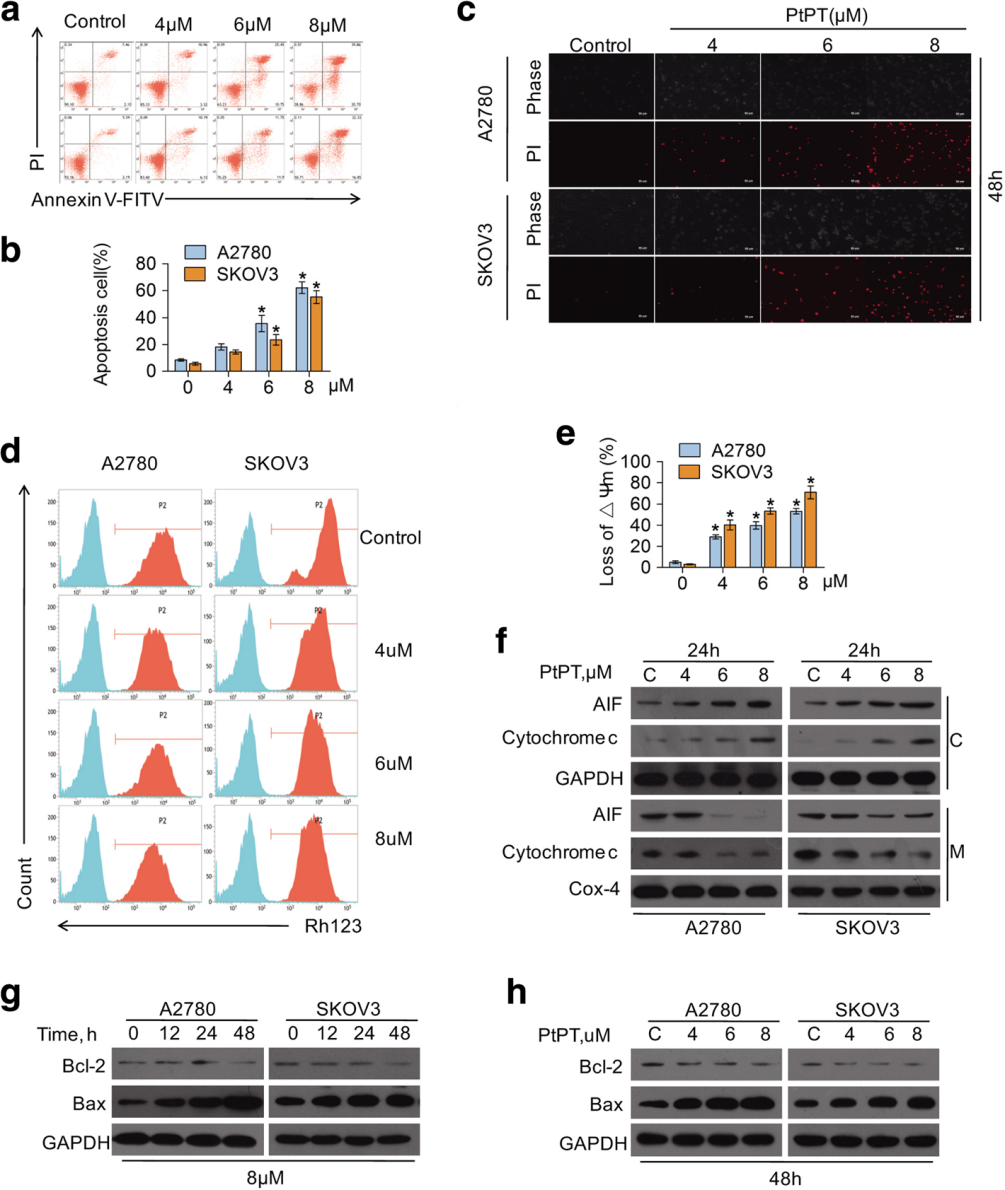
PtPT induces apoptosis in ovarian cancer cells. **a** and **b** A2780 and SKOV3 cells were treated with indicating doses of PtPT for 48 h. Apoptotic cells were detected with Annexin V-FITC and PI double staining followed by flow cytometry (**a**) and summary of cell death (**b**). Mean ± SD (*n* = 3), *P < 0.05. **c** Cells were treated with indicating concentrations of PtPT, the PI-positive cells were recorded under an inverted fluorescence microscope. Representative images were shown. **d** and **e** Cells were treated with 4, 6 and 8 μM PtPT for 24 hours. Mitochondrial membrane potential were detected using rhodamine-123 staining and followed by flow cytometry analysis. Representative images and graph were shown. Mean ± SD (*n* =3), *P < 0.05. **f** A2780 and SKOV3 cells were exposed to indicating concentrations of PtPT for 24 h. The cytosolic and mitochondrial fraction were extracted by digitonin buffer and Mitochondria Isolation Kit, respectively. AIF and cytochrome C were detected with western blot analyses. Cox-4 was used as a loading control for the mitochondrial fraction (C: cytosolic fraction; M: mitochondrial fraction). **g** Cells were treated with 8 μM PtPT at indicating time, and (**h**) with indicating concentrations of PtPT for 24 h. The anti-apoptotic proteins Bcl-2 and pro-apoptotic protein Bax were analyzed by western blot. GAPDH was used as a loading control

We then examined the capacity of PtPT to induce cell death in these two cell lines. A2780 and SKOV3 cells were treated with indicated concentrations of PtPT for 48 h, and then the apoptotic cells were detected respectively by flow cytometry with Annexin V-FITC/PI double staining (Fig. 3a and b). To observe the morphological alteration, the effect on cell death by PtPT was also detected by PI staining with inverted fluorescent microscopy (Fig. 3c). We found that PtPT significantly induced apoptosis of A2780 and SKOV3 cells. It is widely acknowledged that mitochondria are the regulating centers of apoptosis. Release of cytochrome C and AIF from mitochondria to the cytoplasm has been accepted as an indicator in the early stage of apoptosis. As shown in Fig. 3d and e, after treatment with PtPT, the integrity of mitochondrial membranes was obviously compromised in both A2780 and SKOV3 cells. To determine whether PtPT triggers the mitochondrial pathway, cancer cells were exposed to PtPT for 24 h. Cytosolic and mitochondrial fractions were extracted and the cytochrome C and AIF levels were detected by western blot analyses. The results showed that the levels of cytosolic cytochrome C and AIF were elevated, and at the same time, reciprocal levels of mitochondrial cytochrome C and AIF were decreased (Fig. 3f), which confirmed that PtPT activated the mitochondrial apoptosis pathway.

**Fig. 4:**
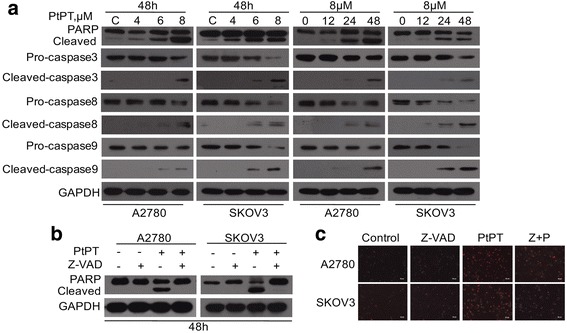
PtPT induces apoptosis in a caspase-dependent manner. **a** A2780 and SKOV3 cells were treated with PtPT at indicating doses for indicating time. PARP and caspase-3,−8,−9 cleavage were detected with western blot. GAPDH was used as a loading control. **b** A2780 and SKOV3 cells were treated with 6 μM PtPT in the presence or absence of caspase inhibitor z-VAD-fmk (50 μM) for 48 h. PARP and caspase-8 were detected with western blot analyses. GAPDH was used as a loading control. **c** Cells treated as (**b**), the PI-positive cells were recorded under an inverted fluorescence microscope. Representative images were shown

Based on the above findings, we sought to detect the expression levels of Bax and Bcl-2 proteins which participate in the endogenous apoptosis pathway (Fig. 3g and h). It is found that PtPT lessened the level of anti-apoptotic proteins Bcl-2 in both A2780 and SKOV3 cells. Along with increasing the level of proapoptotic protein Bax, A2780 and SKOV3 cells were then cultured with PtPT, followed by investigating the caspase activation and PARP cleavage. As shown in Fig. 4a, PtPT dramatically activated caspase-3,−8 and −9 and increased the cleavage of PARP. Furthermore, we found that pan-caspase inhibitor z-VAD-fmk could significantly prevent PtPT-induced cell death (Fig. 4b and c). These data suggested that PtPT triggers A2780 and SKOV3 cell death likely via caspase activation.

### PtPT triggers unfolded protein response (UPR) and accumulation of ubiquitinated proteins

**Fig. 5:**
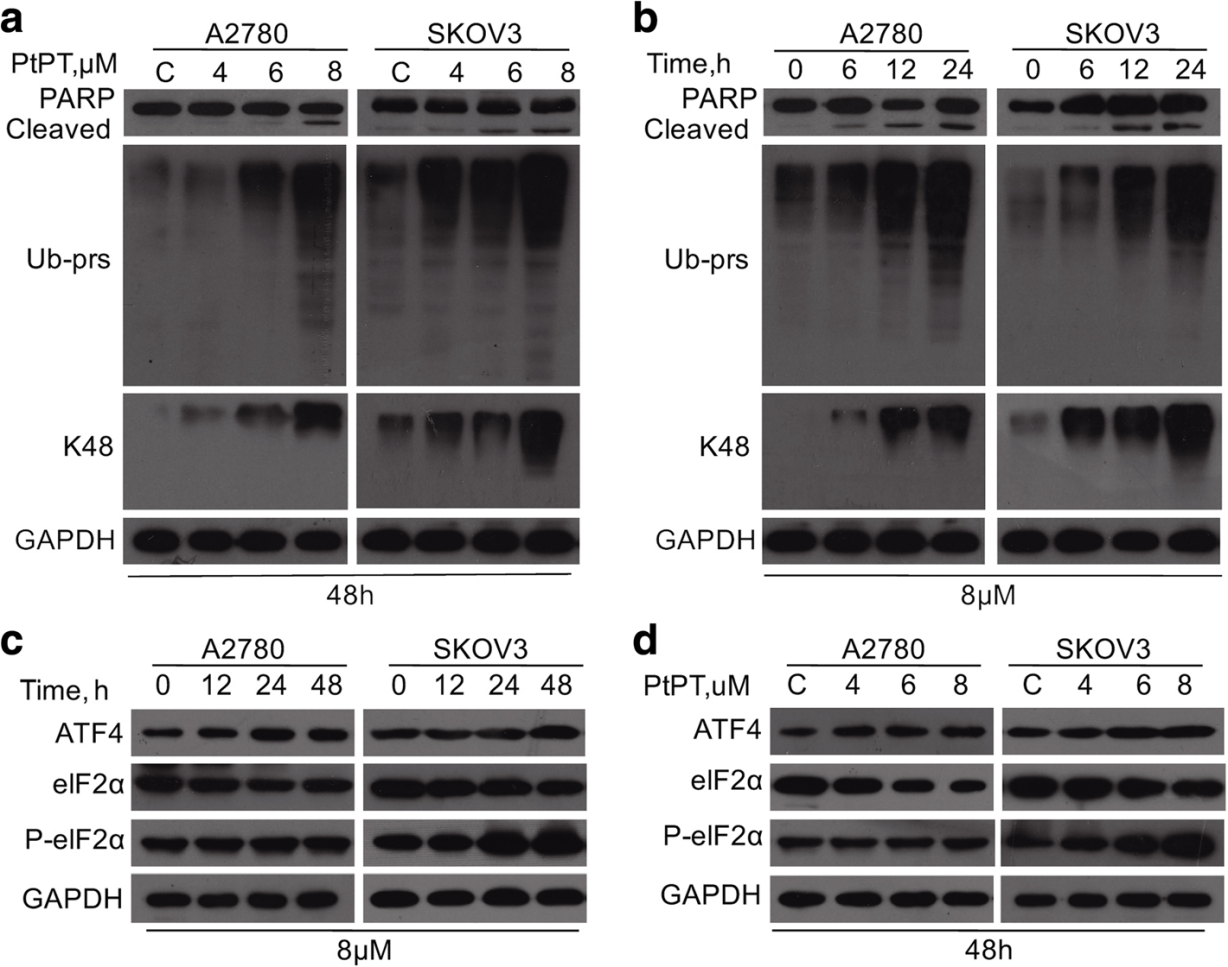
PtPT treatment led to Ub-prs accumulation and ER stress. **a** and **b** A2780 and SKOV3 cells were treated with PtPT at indicating doses for indicating time. PARP, Ub-prs and K48-Ub-prs were detected with western blot. GAPDH was used as a loading control. **c** and **d** A2780 and SKOV3 cells were treated with PtPT at indicating doses for indicating time. ER stress related proteins ATF4, phosphorylated eIF2α (P-eIF2α), and total eIF2α were detected by western blot analysis. GAPDH was used as a loading control

Our previous reports have shown that PtPT induces accumulation of Ub-prs due to inhibition of UCHL-5 and USP14 in other cancer cells [20]. Here we found that PtPT could dose- and time-dependently inhibit proteasome function in both A2780 and SKOV3 cells. We found that PtPT induces accumulation of Ub-prs and PARP cleavage (Fig. 5a). More importantly, 8 μM PtPT treated for 6, 12 or 24 h and the accumulation of Ub-prs was detected during the course of treatment as early as 6 h (Fig. 5b), but PARP cleavage was detected at about 12 h with PtPT treatment (Fig. 5b). These results suggest that the apoptosis by PtPT happens after the proteasome inhibition. These results confirm that PtPT at an early time can obviously inhibit proteasome function in these ovarian cells, related to the induction of cytotoxicity.

We then further evaluated the effects of PtPT on proteasome inhibition-related signal pathways. Several pathways, like ER (endoplasmic reticulum) stress is involved in proteasome inhibition-induced cell death. We found that PtPT treatment increased ATF4 and P-eIF2α expression in a dose and time-dependent manner (Fig. 5c and d), indicating a sustained activation of the UPR. The ER is the main place for protein synthesis, folding, and trafficking, and excessive ER stress will trigger apoptosis. The above results indicate that ER stress may be involved in PtPT- induced cancer cell death.

### PtPT exhibits anti-cancer activity in vivo

**Fig. 6:**
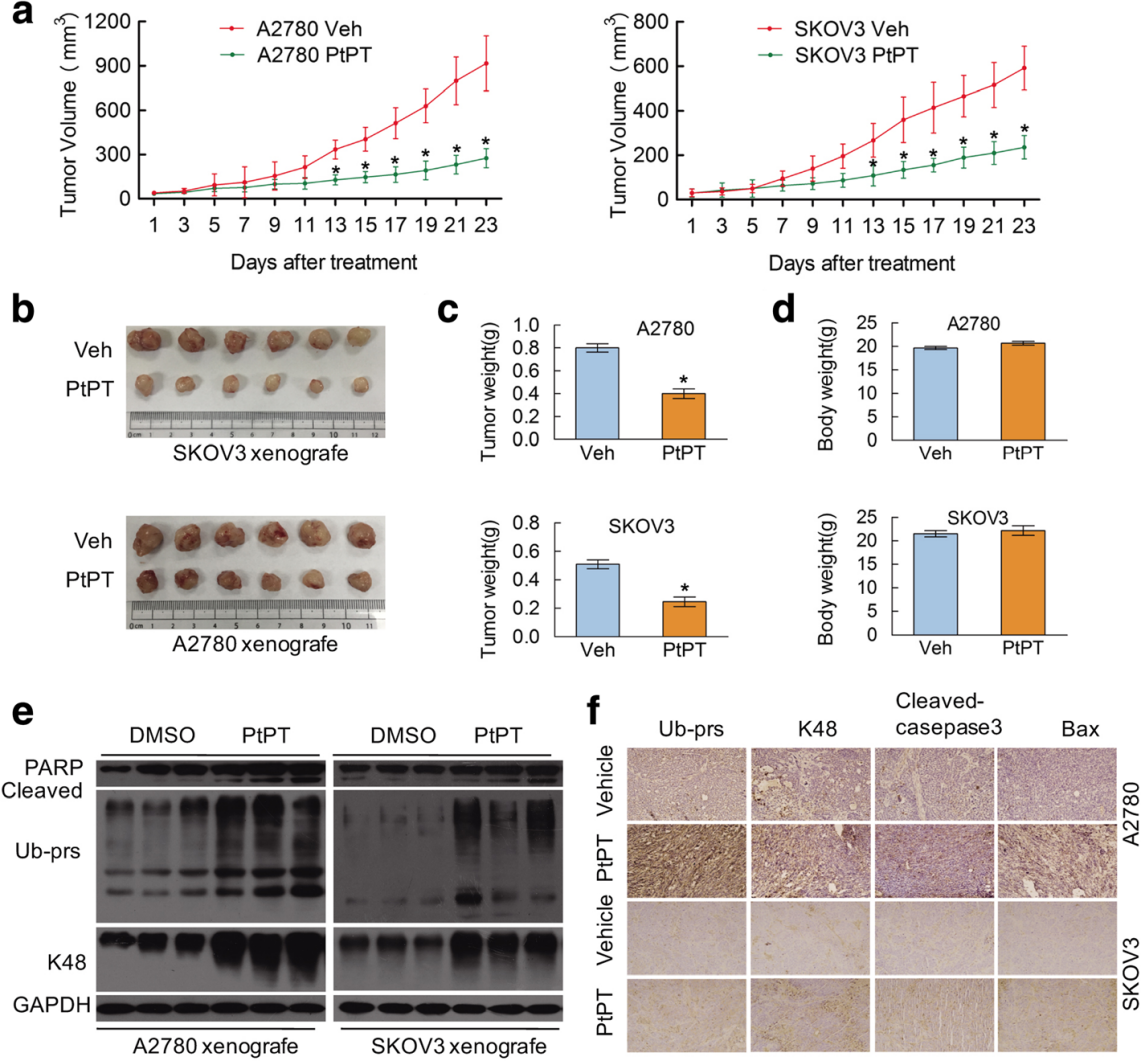
PtPT suppressed ovarian tumor xenografts in nude mice. Nude BALB/c mice bearing A2780 and SKOV3 xenografts were randomized to vehicle and PtPT (7.5 mg/kg/day, i.p.) treatment group. Administration of vehicle or PtPT was initiated when the average tumor size reached at 50 mm^3^ . Tumor size was recorded every other day. Tumor size (**a**), tumor images (**b**), tumor weight (**c**) and body weight (**d**) were shown. Mean ± SD (*n* = 6). *P < 0.05, compared with each control. **e** Total proteins in tumor tissues were extracted and subsequently detected with western blot using PARP, Ub-prs and K48-Ub-prs antibodies. GAPDH was used as a loading control. **f** Representative images of immunohistochemical staining for Ub-prs, K48-Ub-prs, pro-apoptotic protein Bax, and cleaved caspase-3 in tumor tissues from mice bearing A2780 and SKOV3 tumors treated with either vehicle or PtPT (200 ×)

Given that in vitro experiments showed a promising antitumor activity by PtPT on human ovarian cancer cell lines, we next investigated the in vivo effect of PtPT using nude female mouse xenograft models. We found that tumor weight and tumor size of nude mouse models in PtPT treatment group were significantly reduced compared to the control group (Fig. 6a-c) while there were no significant differences in body weight among two groups (Fig. 6d). Western blot analysis showed that PtPT increased the levels of Ub-prs, K48-linked Ub-prs, and cleaved PARP (Fig. 6e). Similarly to the western blot results, the immunostaining analysis was employed to detect the in vivo proteasome function with PtPT. Ub-prs, K48-linked Ub-prs, Bax, and activated caspase-3 proteins were all increased (Fig. 6f) in the PtPT treated group. Together, these results demonstrate that PtPT selectively inhibits proteasome function and tumor growth in vivo. PtPT could selectively inhibit tumor growth in vivo without apparent non-specific toxicity, with a distinct mechanism involving proteasome inhibition.

## Discussion

EOC is the primary reason for death from gynecologic cancer that affects women in the world every year [30]. Although most patients initially show sensitivity to cisplatin, a few years later the drug-resistant metastatic disease occurs [31, 32]. Thus, developing novel anti-tumor therapies that can work alone, or in combination with platinum-based therapy is critical [33].

Bortezomib, the first 20S proteasome peptide inhibitor approved by United States Food and Drug Administration (FDA) for treating multiple myeloma MM , has also been showing its antitumor activity in other malignancies, including colon cancer, prostate cancer, breast cancer, and ovarian cancer [34, 35]. It was reported that ovarian cancer cells may have a greater requirement for proteasomal activity than their untransformed counterpart [36]. Therefore, identifying novel agents that target proteasome, both 19S and 20S, is of great research and clinical value.

Up to date, numerous metals, such as copper, zinc, and gold, have shown their antitumor activity in several carcinomas as proteasome inhibitors. Our previous study also found that PtPT is a specific proteasome inhibitor with low toxicity. However, the effect of PtPT (proteasome-associated deubiquitinase inhibitor) on ovarian cancer cells has not been unraveled. In the current study, we showed that PtPT is an effective chemical against ovarian cancer cells, including A2780 and SKOV3 cells. Additionally, we suggested that PtPT-mediated proteasome inhibition and caspase activation is responsible for PtPT-induced cytotoxicity in ovarian cancer cells. Most importantly, PtPT induced typical proteasome inhibition in both A2780 and SKOV3 cells in vitro and in vivo.

Firstly, we observed that PtPT dramatically suppressed the proliferation of EOC cells, indicating the potential activity for EOC treatment. We further investigated the pattern that PtPT inhibits the EOC proliferation. The ubiquitin-proteasome system is essential in the degradation of several proteins that are involved in the cell cycle control and thereby regulate the growth of cancer cells. Dysregulation of ubiquitin-proteasome system may promote carcinogenesis. We found that PtPT arrested cell cycle progression at G2/M phase in both A2780s and SKOV3 cells via down-regulating Cdc2 and CyclinB1 protein expressions (Fig. 2b and c). It is known that Cyclin B1 is involved in the early events of mitosis by interacting with Cdk1; and Cdc2 (p34) is an M-phase promoting factor that induces entry into mitosis [37–39].

Cell fate is tightly regulated by mitochondria and caspases. Li et al have reported that proteasome inhibition-induced Bax accumulation plays an important role in proteasome inhibition-mediated caspase activation and cell apoptosis [40]. In the current study, we found that PtPT increases pro-apoptotic protein Bax while decreasing anti-apoptotic protein Bcl-2 in a dose- and time-dependent manner. We also observed that various concentrations of PtPT led to the decrease of mitochondrial membrane integrity, thereby inducing the release of cytochrome C and AIF. Similar to previous study, the released apoptotic factors, either directly or by forming a caspase-9 complex, induced caspase activation (Fig. 4). To determine whether caspase activation is required for PtPT-induced cell death, cells were treated with PtPT (6 μM) for 48 hours with pan-caspase inhibitor z-VAD, then PARP was detected by western blot. It was found that PARP cleavage was almost completely rescued in the presence of z-VAD, consistent with the previous reports [23]. PI staining results also showed that z-VAD could reverse PtPT induced cell death (Fig. 4b and c). These results suggest that PtPT-induced A2780 and SKOV3 cell death is associated with caspase activation. Endoplasmic reticulum stress-associated pathways are critical in the treatment of Ovarian Carcinoma [41]. Previous studies have shown that agents including bortezomib, bAP-15, and auranofin that inhibited proteasome and induced Ub-proteins accumulation also induced ER-stress, which may represent a mutual pattern [17, 18]. The current study also found that PtPT significantly induced ER-stress in EOC cells (Fig. 5c and d), suggesting ER-stress may be involved in the PtPT-induced apoptosis.

In addition to our in vitro studies, we also estimated anticancer activity of PtPT in EOC xenografts. We found that PtPT significantly inhibits ovarian cancer growth of EOC xenografts. Importantly, compared with vehicle control, PtPT treatment has no apparent side toxicity, including the reduction of body weight which is commonly seen in CDDP treatment. These results strongly suggest that PtPT is a better anticancer reagent than CDDP and could be used in the treatment of ovarian cancers. As expected, further results confirming that PtPT inhibits the UPS in vivo via targeting proteasomal DUBs. Briefly, PtPT may be an ideal antitumor agent and could be of great value in the treatment of ovarian cancers.

## Conclusion

EOC, an increasing threat to women, remains an incurable carcinoma. Novel chemicals with lower side toxicity are needed for EOC treatment. PtPT significantly inhibited USP14 and UCHL5, thereby accumulating Ub-conjugates. The Ub-conjugates accumulation led to ER stress and caspase activation, which may be the pathway by which PtPT induces apoptosis. Additionally, PtPT induced G2/M phase arrest and thereby dramatically suppressed the proliferation of EOC. In summary, we identified that PtPT, a novel chemical that selectively inhibits 19S proteasomal DUBs USP14 and UCHL5, has potent antitumor activity in EOC without obvious side effect, and could be of great clinical value in the future.

## Abbreviations

AIF: Apoptosis-inducing factor
DMSO: Dimethyl sulfoxide
DUBs: Deubiquitinases
EOC: Epithelial ovarian cancer
ER: Endoplasmic reticulum
PI: Proteasome inhibitor
PtPT: Platinum pyrithione
Ub-prs: Ubiquitinated proteins
UPR: Unfolded protein response
UPS: Ubiquitin proteasome system

## References

[1] Huang W, Zhou Q, Yuan X, Ge ZM, Ran FX, Yang HY, Qiang GL, Li RT, Cui JR (2016). Proteasome Inhibitor YSY01A Enhances Cisplatin Cytotoxicity in Cisplatin-Resistant Human Ovarian Cancer Cells. J Cancer.

[2] Worzfeld T, Pogge von Strandmann E, Huber M, Adhikary T, Wagner U, Reinartz S, Muller R (2017). The Unique Molecular and Cellular Microenvironment of Ovarian Cancer. Front Oncol.

[3] Ferrari F, Bellone S, Black J, Schwab CL, Lopez S, Cocco E, Bonazzoli E, Predolini F, Menderes G, Litkouhi B (2015). Solitomab, an EpCAM/CD3 bispecific antibody construct (BiTE(R)), is highly active against primary uterine and ovarian carcinosarcoma cell lines in vitro. J Exp Clin Cancer Res.

[4] de Melo AC, Paulino E, Garces AH (2017). A Review of mTOR Pathway Inhibitors in Gynecologic Cancer. Oxid Med Cell Longev.

[5] Bruning A, Burger P, Vogel M, Rahmeh M, Friese K, Lenhard M, Burges A (2009). Bortezomib treatment of ovarian cancer cells mediates endoplasmic reticulum stress, cell cycle arrest, and apoptosis. Invest New Drugs.

[6] Johnson DE (2015). The ubiquitin-proteasome system: opportunities for therapeutic intervention in solid tumors. Endocr Relat Cancer.

[7] Huang H, Liao Y, Liu N, Hua X, Cai J, Yang C, Long H, Zhao C, Chen X, Lan X (2016). Two clinical drugs deubiquitinase inhibitor auranofin and aldehyde dehydrogenase inhibitor disulfiram trigger synergistic anti-tumor effects in vitro and in vivo. Oncotarget.

[8] Wang L, Chen YJ, Xu K, Wang YY, Shen XZ, Tu RQ (2014). High expression of UCH37 is significantly associated with poor prognosis in human epithelial ovarian cancer. Tumour Biol.

[9] D’Arcy P, Wang X, Linder S (2015). Deubiquitinase inhibition as a cancer therapeutic strategy. Pharmacol Ther.

[10] Fraile JM, Quesada V, Rodriguez D, Freije JM, Lopez-Otin C (2012). Deubiquitinases in cancer: new functions and therapeutic options. Oncogene.

[11] Pfoh R, Lacdao IK, Saridakis V (2015). Deubiquitinases and the new therapeutic opportunities offered to cancer. Endocr Relat Cancer.

[12] Aressy B, Jullien D, Cazales M, Marcellin M, Bugler B, Burlet-Schiltz O, Ducommun B (2010). A screen for deubiquitinating enzymes involved in the G(2)/M checkpoint identifies USP50 as a regulator of HSP90-dependent Wee1 stability. Cell Cycle.

[13] Joo HY, Zhai L, Yang C, Nie S, Erdjument-Bromage H, Tempst P, Chang C, Wang H (2007). Regulation of cell cycle progression and gene expression by H2A deubiquitination. Nature.

[14] Typas D, Luijsterburg MS, Wiegant WW, Diakatou M, Helfricht A, Thijssen PE, van den Broek B, Mullenders LH, van Attikum H (2015). The de-ubiquitylating enzymes USP26 and USP37 regulate homologous recombination by counteracting RAP80. Nucleic Acids Res.

[15] Wei R, Liu X, Yu W, Yang T, Cai W, Liu J, Huang X, Xu GT, Zhao S, Yang J, Liu S (2015). Deubiquitinases in cancer. Oncotarget.

[16] D’Arcy P, Brnjic S, Olofsson MH, Fryknas M, Lindsten K, De Cesare M, Perego P, Sadeghi B, Hassan M, Larsson R, Linder S (2011). Inhibition of proteasome deubiquitinating activity as a new cancer therapy. Nat Med.

[17] Liu N, Li X, Huang H, Zhao C, Liao S, Yang C, Liu S, Song W, Lu X, Lan X (2014). Clinically used antirheumatic agent auranofin is a proteasomal deubiquitinase inhibitor and inhibits tumor growth. Oncotarget.

[18] Tian Z, D’Arcy P, Wang X, Ray A, Tai YT, Hu Y, Carrasco RD, Richardson P, Linder S, Chauhan D, Anderson KC (2014). A novel small molecule inhibitor of deubiquitylating enzyme USP14 and UCHL5 induces apoptosis in multiple myeloma and overcomes bortezomib resistance. Blood.

[19] Fornaro L, Vivaldi C, Cereda S, Leone F, Aprile G, Lonardi S, Silvestris N, Santini D, Milella M, Caparello C (2015). Second-line chemotherapy in advanced biliary cancer progressed to first-line platinum-gemcitabine combination: a multicenter survey and pooled analysis with published data. J Exp Clin Cancer Res.

[20] van Haaften C, Boot A, Corver WE, van Eendenburg JD, Trimbos BJ, van Wezel T (2015). Synergistic effects of the sesquiterpene lactone, EPD, with cisplatin and paclitaxel in ovarian cancer cells. J Exp Clin Cancer Res.

[21] Rabik CA, Dolan ME (2007). Molecular mechanisms of resistance and toxicity associated with platinating agents. Cancer Treat Rev.

[22] Wang J, Wu GS (2014). Role of autophagy in cisplatin resistance in ovarian cancer cells. J Biol Chem.

[23] Zhao C, Chen X, Zang D, Lan X, Liao S, Yang C, Zhang P, Wu J, Li X, Liu N (2016). Platinum-containing compound platinum pyrithione is stronger and safer than cisplatin in cancer therapy. Biochem Pharmacol.

[24] Huang H, Hua X, Liu N, Li X, Liu S, Chen X, Zhao C, Lan X, Yang C, Dou QP, Liu J (2014). Anacardic acid induces cell apoptosis associated with induction of ATF4-dependent endoplasmic reticulum stress. Toxicol Lett.

[25] Liao Y, Liu N, Hua X, Cai J, Xia X, Wang X, Huang H, Liu J (2017). Proteasome-associated deubiquitinase ubiquitin-specific protease 14 regulates prostate cancer proliferation by deubiquitinating and stabilizing androgen receptor. Cell Death Dis.

[26] Huang H, Liu N, Yang C, Liao S, Guo H, Zhao K, Li X, Liu S, Guan L, Liu C (2012). HDAC inhibitor L-carnitine and proteasome inhibitor bortezomib synergistically exert anti-tumor activity in vitro and in vivo. PLoS One.

[27] Shi X, Chen X, Li X, Lan X, Zhao C, Liu S, Huang H, Liu N, Liao S, Song W (2014). Gambogic acid induces apoptosis in imatinib-resistant chronic myeloid leukemia cells via inducing proteasome inhibition and caspase-dependent Bcr-Abl downregulation. Clin Cancer Res.

[28] Huang H, Chen D, Li S, Li X, Liu N, Lu X, Liu S, Zhao K, Zhao C, Guo H (2011). Gambogic acid enhances proteasome inhibitor-induced anticancer activity. Cancer Lett.

[29] Shi X, Lan X, Chen X, Zhao C, Li X, Liu S, Huang H, Liu N, Zang D, Liao Y (2015). Gambogic acid induces apoptosis in diffuse large B-cell lymphoma cells via inducing proteasome inhibition. Sci Rep.

[30] Jemal A, Siegel R, Ward E, Hao Y, Xu J, Murray T, Thun MJ (2008). Cancer statistics, 2008. CA Cancer J Clin.

[31] Kikkawa F, Nawa A, Ino K, Shibata K, Kajiyama H, Nomura S (2006). Advances in treatment of epithelial ovarian cancer. Nagoya J Med Sci.

[32] Mandato VD, Abrate M, De Iaco P, Pirillo D, Ciarlini G, Leoni M, Comerci G, Ventura A, Lenzi B, Amadori A (2013). Clinical governance network for clinical audit to improve quality in epithelial ovarian cancer management. J Ovarian Res.

[33] Malm SW, Hanke NT, Gill A, Carbajal L, Baker AF (2015). The anti-tumor efficacy of 2-deoxyglucose and D-allose are enhanced with p38 inhibition in pancreatic and ovarian cell lines. J Exp Clin Cancer Res.

[34] Kao C, Chao A, Tsai CL, Chuang WC, Huang WP, Chen GC, Lin CY, Wang TH, Wang HS, Lai CH (2014). Bortezomib enhances cancer cell death by blocking the autophagic flux through stimulating ERK phosphorylation. Cell Death Dis.

[35] Nawrocki ST, Sweeney-Gotsch B, Takamori R, McConkey DJ (2004). The proteasome inhibitor bortezomib enhances the activity of docetaxel in orthotopic human pancreatic tumor xenografts. Mol Cancer Ther.

[36] Bazzaro M, Lee MK, Zoso A, Stirling WL, Santillan A, Shih Ie M, Roden RB (2006). Ubiquitin-proteasome system stress sensitizes ovarian cancer to proteasome inhibitor-induced apoptosis. Cancer Res.

[37] Chang CC, Hung CM, Yang YR, Lee MJ, Hsu YC (2013). Sulforaphane induced cell cycle arrest in the G2/M phase via the blockade of cyclin B1/CDC2 in human ovarian cancer cells. J Ovarian Res.

[38] Choi YH, Yoo YH (2012). Taxol-induced growth arrest and apoptosis is associated with the upregulation of the Cdk inhibitor, p21WAF1/CIP1, in human breast cancer cells. Oncol Rep.

[39] Matthess Y, Raab M, Sanhaji M, Lavrik IN, Strebhardt K (2010). Cdk1/cyclin B1 controls Fas-mediated apoptosis by regulating caspase-8 activity. Mol Cell Biol.

[40] Li B, Dou QP (2000). Bax degradation by the ubiquitin/proteasome-dependent pathway: involvement in tumor survival and progression. Proc Natl Acad Sci U S A.

[41] Chuffa LG, Lupi Junior LA, Seiva FR, Martinez M, Domeniconi RF, Pinheiro PF, Dos Santos LD, Martinez FE (2016). Quantitative Proteomic Profiling Reveals That Diverse Metabolic Pathways Are Influenced by Melatonin in an in Vivo Model of Ovarian Carcinoma. J Proteome Res.

